# Symbiosis Specificity of the Preceding Host Plant Can Dominate but Not Obliterate the Association Between Wheat and Its Arbuscular Mycorrhizal Fungal Partners

**DOI:** 10.3389/fmicb.2018.02920

**Published:** 2018-11-27

**Authors:** Catarina Campos, Mário Carvalho, Clarisse Brígido, Michael J. Goss, Tânia Nobre

**Affiliations:** ^1^Instituto de Ciências Agrárias e Ambientais Mediterrânicas, Instituto de Investigação e Formação Avançada, Universidade de Évora, Évora, Portugal; ^2^School of Environmental Sciences, University of Guelph, Guelph, ON, Canada

**Keywords:** arbuscular mycorrhizal fungi symbiosis, extraradical mycelium, host–symbiont specificity, host plant transition, soil disturbance, symbiosis-related genes, *Triticum aestivum* L.

## Abstract

The symbiosis established between arbuscular mycorrhizal fungi (AMF) and roots of most land plants plays a key role in plant nutrient acquisition and alleviation of environmental stresses. Despite the ubiquity of the symbiosis, AMF and host species display significant specificity in their interactions. To clarify preferential associations between wheat (*Triticum aestivum*) and AMF, we characterized root AMF communities in the transition from two first host species, ryegrass (*Lolium rigidum*) and yellow-serradella (*Ornithopus compressus*), grown separately or together, to a second host (wheat), by sequencing the large subunit ribosomal DNA (LSU rDNA) gene. The response of AMF communities in wheat to prior soil disturbance – and consequently of the mycelial network [intact extraradical mycelium (ERM) vs. disrupted mycelium] established with either of the first hosts – was also investigated. Since the outcome of a specific host–symbiont interaction depends on the molecular responses of the host plant upon microbial colonization, we studied the expression of six key symbiosis-related genes in wheat roots. AMF communities on *L. rigidum* and *O. compressus* roots were clearly distinct. Within an undisturbed ERM, wheat AMF communities were similar to that of previous host, and *O. compressus*-wheat-AMF interactions supported a greater growth of wheat than *L. rigidum*-wheat-AMF interactions. This effect declined when ERM was disrupted, but generated a greater activation of symbiotic genes in wheat, indicating that plant symbiotic program depends on some extent on the colonizing symbiont propagule type. When a mixture of *L. rigidum* and *O. compressus* was planted, the wheat colonization pattern resembled that of *O. compressus*, although this was not reflected in a greater growth. These results show a lasting effect of previous hosts in shaping wheat AMF communities through an efficient use of the established ERM, although not completely obliterating host–symbiont specificity.

## Introduction

It is strongly held that the symbiosis with arbuscular mycorrhizal fungi (AMF) was a major driver of colonization of land by plants and fundamentally altered environmental conditions on earth (Humphreys et al., 2010). Amongst the plant microbiome, the interaction with AMF is one of the most important symbiotic associations. AMF communities can enhance plant diversity and productivity of natural ecosystems (Van Der Heijden et al., 1998; Klironomos et al., 2000) and affect multiple functions and processes, from nutrient uptake to drought and soil metal stress alleviation (Brito et al., 2014; Yang et al., 2016; Huang et al., 2017; Sharma et al., 2017). The symbionts are horizontally transmitted (through mycelial networks) and individual plants can simultaneously associate with multiple species of AMF that may differ in provision and delivery of benefits.

Despite their ubiquity and ability to colonize most plants, AMF can exhibit considerable selectivity in their association with different host species, and *vice-versa* (Helgason and Fitter, 2009; Öpik et al., 2009). Coexisting plant species can harbor distinct AMF communities (Öpik et al., 2009; Hazard et al., 2013; Davison et al., 2015); this is not surprising since in common with their hosts, arbuscular mycorrhiza differ in life-history traits and some host–symbiont combinations can be more beneficial than others (Bever, 2002; Zhang et al., 2010; Walder et al., 2012). Therefore, the functional attributes of both partners seem to relate to the strength or weakness of the host–symbiont interaction. In addition to the specific host plant genotype, there is also a strong relation between composition and richness of AMF communities in a particular plant species and the structure of the neighboring plant communities (Hausmann and Hawkes, 2009; Kohout et al., 2015). The closeness of different plant species harboring distinct AMF communities can increase both species richness and induce shifts in the AMF root community composition of a particular host (Zhang et al., 2010; Meadow and Zabinski, 2012).

The delivery of AMF-induced benefits to crop plants is of particular interest for agricultural crop production and sustainability. However, it is clear that a well-established AMF extraradical mycelial (ERM) network is crucial for an adequate degree of benefit delivery (Kabir, 2005; Brito et al., 2012). Brito et al. (2014) reported that a greater growth of wheat (*Triticum aestivum* L.) in a soil with excessive levels of manganese occurred when yellow-serradella (*Ornithopus compressus* L.) rather than ryegrass (*Lolium rigidum* Gaudin) was grown prior to wheat, provided that the soil was undisturbed and therefore an intact ERM was present when wheat was planted. More recently, Brígido et al. (2017) reported that wheat grown in undisturbed soil after *O. compressus* acquired an AMF community closely related to that of the previous host plant, and different to the one found when soil was disturbed or not cropped prior to planting wheat. These findings indicated that the host relation with the inoculum source (intact ERM or disrupted mycelium) is dynamic and important for plant performance. Furthermore, the outcome of a specific host–symbiont interaction depends on the dynamic molecular responses that the host plant set up upon microbial colonization. For example, closely related AMF have been shown to alter transcriptional profiles of *Medicago trunculata* (Hohnjec et al., 2005). The greater growth of wheat after undisturbed *O. compressus* than after *L. rigidum* seems, therefore, to indicate molecular specificities of the symbiosis.

In this context, the present study explores the composition of indigenous AMF assemblages in plant roots in a transition from a Poaceae (*L. rigidum*) or a Fabaceae (*O. compressus*) to a different Poaceae (wheat). Since mixed planting occurs universally in natural and cultivated ecosystems, the effect of co-planting two host species was also included in this study. We hypothesize that: (i) there are preferential associations between AMF and host species; however, these mutual preferences are modified when two plant species grow together (neighbor effect); (ii) there is an influence of the AMF symbiosis established with the first host species on the succeeding symbiosis formed with the second host (wheat); and (iii) the disturbance state of the soil and consequently of the established mycelial network with the first host plants has an influence on the AMF communities of the succeeding plant and on the regulation of key symbiosis-related genes [*CASTOR*, *POLLUX*, *CCaMK*, *Cyclops*, *SCL26* (*NSP2*), and *PhT1* *Myc*]. Our experimental design allowed us to investigate symbiont-host preferences at contrasting levels of soil disturbance and relative AMF composition of the mycelial network.

## Materials and Methods

### Biological Samples

Biological material was obtained through a two-phase greenhouse experiment designed to evaluate the AMF symbiotic community associated with two first host plant species, and after the transition to a second host species (wheat). We used an unsterilized sandy loam Eutric Cambisol soil obtained from a natural pasture, as described by Brito et al. (2014).

In phase 1 of the experiment, two different mycotrophic plant species (Poaceae *L. rigidum* and Fabaceae *O. compressus*) (Goss et al., 2017) were grown in 8 L pots over 8 weeks, either individually or as a mixture of both species (the *O. compressus* system, the *L. rigidum* system and the mixture system). Six replicate pots were used per system, with three plants per pot in the case of the individual species and six in the case of the mixture (three plants of each species). Plants were grown under the natural light/dark regime. At the end of phase 1, we excised the plant shoots. In half of the replicates of each system (three pots) we passed the soil through a 4 mm sieve, thus creating an AMF source comprising spores and fragmented extraradical mycelium (ERM) (disturbed treatment). In the other half (three pots), soil was kept undisturbed and consequently the mycelial network remained intact (undisturbed treatment). Samples of soil from the disturbed treatments were taken from each pot (total of nine samples), dried and cleared of root fragments. Root samples of *O. compressus* and *L. rigidum* were also taken from each pot where soil was disturbed: three samples from *L. rigidum* (hereafter designated as Lol), three from *O. compressus* (hereafter designated as Orn), three from *L. rigidum* from the mixture (hereafter designated as LolMix) and three samples from *O. compressus* from the mixture (hereafter designated as OrnMix). Roots were washed, dried at 50°C during 24 h, crushed using liquid nitrogen and kept at -80°C for later DNA extraction.

In phase 2 of the experiment, wheat (Family: Poaceae, *T. aestivum* L., var. Ardila) seedlings were planted in the same pots as before (three plants per pot). Living plants of *L. rigidum* or *O. compressus* were never present during this second phase of the experiment, only roots or root fragments remained (for undisturbed and disturbed treatments, respectively). Therefore the wheat treatments were as following: wheat after *O. compressus* undisturbed (hereafter designated as WOU), wheat after *O. compressus* disturbed (hereafter designated as WOD), wheat after *L. rigidum* undisturbed (hereafter designated as WLU), wheat after *L. rigidum* disturbed (hereafter designated as WLD), wheat after the mixture undisturbed (hereafter designated as WMixU), wheat after the mixture disturbed (hereafter designated as WMixD). Wheat was allowed to grow for 5 weeks and then root samples were taken. Roots were cleaned, washed in distilled water, dried at 50°C during 24 h, crushed in liquid nitrogen and stored at -80°C for further DNA extractions. For RNA extraction, root samples were washed in distilled water, snap-frozen in liquid nitrogen and stored at -80°C. For each individual plant, fresh weight of the shoot was measured. Significant differences in wheat shoot fresh weight between the different treatments (with disturbance and preceding host as factors) were determined by two-way ANOVA using the R package ‘stats.’

### DNA Extraction and Sequencing

Genomic DNA was extracted from each root sample with the DNeasy Plant Mini Kit (Qiagen, Hilden, Germany) according to the manufacturer’s instructions. DNA from soil samples was extracted with ZR Soil Microbe DNA MicroPrep^TM^ (Zymo Research, Irvine, CA, United States) according to the manufacturer’s instructions. The quality and concentration of the DNA extracted were assessed using a NanoDrop 2000-C spectrophotometer (Thermo Scientific, Wilmington, DE, United States).

DNA samples of soil and roots were prepared for Illumina MiSeq sequencing technology with 2 bp × 250 bp chemistry in a two-step nested polymerase chain reaction (PCR) procedure. The specific eukaryotic primers LR1 and NDL22 (Van Tuinen et al., 1998) were used to amplify a fragment of the large ribosomal subunit (LSU) DNA (rDNA) genes in the first PCR, whereas the primers FRL3 and FRL4 (Gollotte et al., 2004) were used to amplify the LSU-D2 rDNA genes of AMF in the second PCR. To identify each sample, the primers FRL3 and FRL4 were tagged with a 10-mer multiplex identifier sequence at the 5′ end of the primers. At least three technical replicates per sample were used in each step. Further preparatory procedures, such as equimolar pooling of PCR reactions, addition of adaptors and PCR product purification were performed by GenoScreen^1^, prior to Illumina sequencing.

Illumina MiSeq sequencing yielded 3,542,923 raw sequences. Raw sequence data were processed using the CLC Genomics Workbench version 8.5.1. (Qiagen Aarhus A/S, Denmark). Data were sorted by index and forward and reverse reads merged for each of the samples. Sequences were trimmed to a minimum length of 300 bp and reads with low coverage removed from subsequent analyses. The remaining sequence reads were checked for chimeric sequences using the CLC Genomics Workbench (26,322 chimeric sequences were discarded), *de novo* clustered at 97% similarity threshold and assigned to operational taxonomic units (OTUs). OTU abundance tables were created using the clustered sequences. Affiliation to AMF was checked by BLAST against the public databases DDBJ/EMBL/GenBank and MaarjAM (Öpik et al., 2010). Raw sequencing data were submitted to the Sequence Read Archive under the accession SRP128462.

### Sequencing Statistical Analysis

To evaluate sampling effort, a rarefaction analysis of data subsets was done using the function *rarecurve* from the R package ‘vegan’ (Oksanen et al., 2016). However, to compare AMF communities between samples regardless of sampling depth artifacts, the number of sequences per sample was normalized down to that of the sample with the lowest number of reads through a random selection of sequences in each sample, using the function *rrarefy* from the R package ‘vegan’ (Oksanen et al., 2016). The effect of data normalization on the variation in AMF communities’ composition was evaluated by repeating the analyses with the non-standardized data.

Permutational multivariate analysis of variance (PERMANOVA) (Anderson and Walsh, 2013) with the Bray–Curtis dissimilarity as a measure of distance between AMF communities, was performed using the *adonis* routine of the ‘vegan’ package (Oksanen et al., 2016). PERMANOVA was used to address the partition variance in AMF community composition in soil after different host species (*O. compressus*, *L. rigidum* or mixture of both), and to assess AMF variance between first hosts and its putative neighbor effect (*O. compressus*, *L. rigidum*, *O. compressus* neighbored by *Lolium*, and *L. rigidum* neighbored by *Ornithopus*).

Arbuscular mycorrhizal fungi β-diversity among *O. compressus* and *L. rigidum* was analyzed by testing homogeneity of OTU dispersion among the four different groups: Orn, Lol, OrnMix and LolMix. β-Diversity, here defined as the dissimilarity in species composition between hosts, was partitioned following Baselga (2010) in “turnover or replacement” and “nestedness or species loss.” We used OTU’s abundances based on Bray–Curtis dissimilarity index (Bray and Curtis, 1957) because quantitative data has been shown to provide more useful information on the mechanisms shaping diversity patterns within and amongst communities (Ulrich and Gotelli, 2010; Baselga, 2013). Overall and partitioned β-diversity were calculated using the functions *bray.part*, *betadisper*, and *permutest.betadisper* from the ‘vegan’ and ‘betapart’ packages (Oksanen et al., 2016; Baselga et al., 2017).

To test the independent and interacting effects of disturbance and previous host species on AMF β-diversity in wheat, we performed PERMANOVA using the function *adonis* from the R package “vegan.” For that, we partitioned β-diversity into its two components and the output of this partitioning approach consists of three distance matrices representing: (1) β-diversity— i.e., Bray–Curtis; (2) the nestedness component; and (3) the turnover component. The obtained *R*^2^ values quantify the explanatory power of the factors (disturbance, first host and their interaction). Non-metric multidimensional scaling (NMDS) was also used to represent AMF community variation amongst species/treatments in two-dimensional space using the function *metaMDS* from the R package ‘vegan.’

The number of unique and shared OTUs between different treatments/plant species was found using the function *vennDiagram* from the R package ‘VennDiagram’ (Chen and Boutros, 2011).

To detect the specific OTUs differentially abundant between first hosts and wheat plants, we used the *metastats* command based on the Metastats program (White et al., 2009) using 1000 permutations, implemented in the Mothur platform (Schloss et al., 2009). Indicator AMF taxa for first host species were determined using the *indVal* function of the ‘labdsv’ package of R (Roberts, 2012).

Significant differences in the frequencies of shared OTUs grouped by genera between hosts/neighbor effect/disturbance were determined by two-way ANOVA or *t*-test using the R package ‘stats.’

### Wheat Symbiosis-Related Genes Analysis by Quantitative Real Time RT-PCR (qPCR)

Six key plant genes involved in the symbiotic process between AMF were chosen to investigate if wheat showed a differentiated response to previous AMF host and to ERM disturbance: *TaCASTOR*, *TaPOLLUX*, *TaCCaMK*, *TaCyclops*, *TaSCL26* (*NSP2*), and *TaPhyT1* *Myc*. *CASTOR* and *POLLUX* are twin cation channel-enconding genes acting upstream of calcium spiking (Kistner et al., 2005; Chen et al., 2009). The Ca^2+^ ions then bind to a calcium and calmodulin-dependent kinase (CCaMK) leading to the activation of Cyclops, regulating arbuscule formation (Yano et al., 2008; Miller et al., 2013). Downstream of this signaling pathway are the genes regulating the transcriptional outputs, including the GRAS transcription factors. The scarecrow-like protein 26/nodulation signaling pathway (SCL26/NSP2) belongs to the GRAS family and has been found to facilitate mycorrhizal colonization (Maillet et al., 2011; Gobbato et al., 2012). Given that disturbed or undisturbed ERM affects how early and fast the colonization occurs (Goss and De Varennes, 2002; Brito et al., 2014; Alho et al., 2015), we also looked at the expression of the specific AMF induced phosphate transporter *PhT1* Myc gene. Gene sequences were retrieved from Ensembl Plants database. Glyceraldehyde-3-phosphate dehydrogenase (*GAPDH*) and *Ta54227* were used as endogenous reference genes to normalize target gene expression (Paolacci et al., 2009). For each gene, specific primers were designed to span at least one intron/exon border to avoid amplification of potential contaminating genomic DNA, and then its quality analyzed with Netprimer^2^.

Total RNA extraction was performed on individual roots (three plants per pot, three pots per treatment) using the Maxwell^®^ 16 LEV simplyRNA purification kit (Promega, Madison, WI, United States), according to the manufacturer’s instructions. RNA was quantified using a NanoDrop-2000C spectrophotometer (Thermo Scientific) and its integrity was checked by gel electrophoresis. First strand cDNA was synthesized with 500 ng of purified total RNA using the GoScript^TM^ Reverse Transcription System (Promega) using random decamer primers, following manufacturer’s instructions.

Quantification of gene expression was performed by RT-qPCR using 5 μl of 10x diluted cDNA with the Maxima SYBR Green/ROX qPCR Master Mix (2x) (Thermo Scientific) on a 7500 Real Time PCR System (Applied Biosystems, Foster City, CA, United States). RT-qPCR was conducted as described elsewhere (Campos et al., 2016). Primer sequences, amplicon sizes and qPCR amplification efficiencies are shown in Supplementary Table S1. Evaluation of expression stability for the reference genes was done using the statistical application *geNorm* (Vandesompele et al., 2002). Expression of target genes was evaluated by relative quantification using the geometric normalization factors obtained from *geNorm*. Differences in gene expression were analyzed by *t*-test and Nested ANOVA, using pot as random factor and disturbance and previous host as fixed factors, using the R packages ‘nlme’ and ‘multcomp.’

## Results

### Illumina MiSeq Data Analysis

From the 9 soil and 30 root samples analyzed, a total of 238,163 high quality and filtered sequences of the LSU rDNA were obtained. After clustering at 97% sequence identity threshold, all the sequences corresponded to 92 representative distinct OTUs. BLAST searches assigned all sequences to phylum Glomeromycotina (Spatafora et al., 2016) (see Supplementary Table S2 for details regarding % coverage and identity), in spite of the previously noted lack of exclusivity of the used nested primer set (which seem to also amplify some basidiomycetes (Mummey and Rillig, 2008)). These representative sequences were deposited in GenBank (Accession Nos. MG774934 to MG775025). Based on the rarefaction analysis, the number of sequences obtained provided adequate coverage of the AMF diversity in soil and root samples, as curves reached an asymptote (Supplementary Figure S1).

AMF showed a wide diversity, particularly in root samples (Supplementary Table S2). The sequences from all samples were distributed taxonomically amongst six different families within Glomeromycotina, varying greatly in their frequencies: Glomeraceae (65.6%), Claroideoglomeraceae (29.0%), Acaulosporaceae (5.2%), Gigasporaceae (0.2%), Archaeosporaceae (0.03%) and Paraglomeraceae (0.01%). Within the Glomeraceae (55 OTUs), 17 representative sequences (30.9%) were assigned to the genus *Rhizophagus*, 4 to *Funneliformis* (7.3%) and 1 to *Dominikia* (1.8%). The remaining 33 OTUs (60% of the total) had no match in the databases at genus level, thus being reported as uncultured Glomeraceae (Supplementary Table S2).

### General AMF Community Composition in Soil and Roots of Ryegrass and Yellow-Serradella

In the soil and first host species, AMF richness varied from 4 to 45 OTUs, with the smallest values observed in the soil and largest in *O. compressus* (Supplementary Table S2), having average values of 7 OTUs for soil, 29 for *L. rigidum* as standalone species and 27 in the mixture with *O. compressus*, 40 OTUs for *O. compressus* as standalone species and 38 in the mixture with *L. rigidum*.

The most abundant taxa in soil samples matched with uncultured Glomeraceae (∼78%), followed by *Claroideoglomus* (∼20%) and *Rhizophagus* genus (∼2%). *Acaulospora*, *Archaeospora*, *Scutellospora*, and *Paraglomus* comprised less than 1% (Supplementary Figure S2a). In roots of *O. compressus* and *L. rigidum* the most abundant sequences also belonged to uncultured Glomeraceae (50 and 59%, respectively), but *Claroideoglomus* was much more abundant in ryegrass (∼27%) than in yellow-serradella (∼11%). *Acaulospora* was the opposite, being 23% in *Ornithopus* and less than 3% in *Lolium*. *Funneliformis*, *Scutellospora*, *Archaeospora*, and *Paraglomus* sequences comprised less than 2% in both hosts.

**FIGURE 1:**
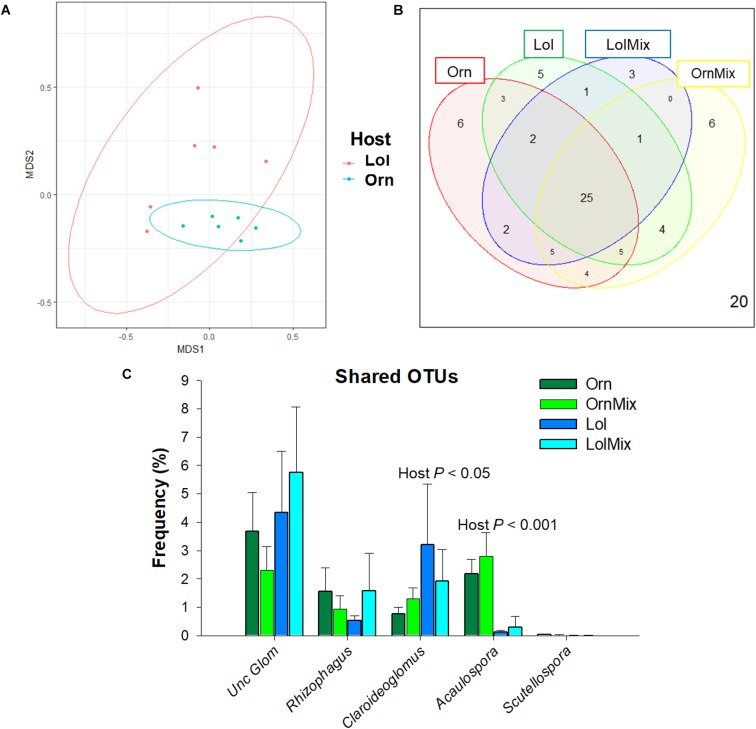
Shared and unique OTUs between *Ornithopus compressus* and *Lolium rigidum*. **(A)** Representation of OTU composition in all *O. compressus* and all *L. rigidum* plants in a multidimensional space (NMDS plot), showing that they cluster depending on host. Ellipses represent ordination confidence intervals (95%) (stress = 0.13). **(B)** Venn diagram representing the number of unique and shared OTUs between the different hosts. **(C)** Frequencies of shared arbuscular mycorrhizal fungi (AMF) taxa grouped by genera in the different hosts. Significant differences between hosts for the same AMF genus (*N* = 3) are indicated. In all cases: Orn, *O. compressus* alone; OrnMix, *O. compressus* neighbored by *L. rigidum*; Lol, *L. rigidum* alone; LolMix, *L. rigidum* neighbored by *O. compressus*. OTUs with no match in the databases at genus level were considered as uncultured Glomeraceae.

Seventy two OTUs were found in *O. compressus* and *L. rigidum*, of which 25 were shared by the four experimental groups: Orn, Lol, OrnMix, and LolMix (Figures 1B,C). The majority of the shared OTUs fell within the uncultured Glomeraceae match (48% of the 72 OTUs), followed by the *Claroideoglomus* genus with around 22% and then by *Acaulospora* (16%). *Rhizophagus* sequences were 14% and *Scutellospora* less than 1%. *O. compressus* had a slightly larger number of unique OTUs (6) than *L. rigidum* (5), and both species showed a slightly greater diversity of AMF genera when in the neighboring of each other (Figure 1B and Supplementary Figure S3a).

The PERMANOVA on the sequencing data from soil DNA did not show any significant differences between soil samples surrounding *O. compressus* or *L. rigidum* (*F* = 0.45, *R^2^* = 0.13, *P* > 0.05). However, AMF community structure between soil and these two hosts was significantly different (PERMANOVA, *F* = 5.82, *R^2^* = 0.23, *P* < 0.01). This was further corroborated by NMDS ordination (Supplementary Figure S2b), which was also consistent with the differences in AMF richness described above.

PERMANOVA analysis revealed that AMF communities were significantly different between *O. compressus* and *L. rigidum* (*F* = 3.47, *R^2^* = 0.25, *P* < 0.001) (Figure 1A); however, there was no significant plant neighboring effect on AMF communities (PERMANOVA *F* = 0.75, *R^2^* = 0.05, *P* > 0.05), indicating that co-planting did not influence AMF communities in these two species.

*Claroideoglomus* abundance was greater in *L. rigidum* than in *O. compressus* (Two-way ANOVA, *P* < 0.05) (Figure 1C). Also, *Acaulospora* frequency was greatly influenced by host (Two-way ANOVA, *P* < 0.001), being much higher in *O. compressus*, regardless whether it was grown alone or in co-planting (Figure 1C). Unique OTUs belonging to *Acaulospora* were found in *O. compressus*, and unique OTUs of *Claroideoglomus* were found in *L. rigidum* (Supplementary Figure S3). These results of non-random association were further supported by the IndVal index, since four *Acaulospora* OTUs were clear indicators of *O. compressus* and one *Claroideoglomus* OTU was an indicator for *L. rigidum* (Supplementary Table S3).

### Wheat Growth Performance and AMF Dynamics in Host Transition

Shoot and root fresh weight of wheat was significantly different between disturbance regimes for all host combinations, being much greater in the undisturbed one (*P* < 0.001) (Supplementary Table S4). Within the undisturbed regime, shoot weight of WOU was significantly greater (1.31 ± 0.33 g, mean ± SD) than weight of WLU (0.96 ± 0.19 g) or WMixU (0.97 ± 0.20 g) (*P* < 0.05) (Supplementary Table S4). Root weight of WOU was significantly greater (0.45 ± 0.20 g) than weight of WMixU (0.25 ± 0.11 g) (*P* < 0.05) (Supplementary Table S4). No significant differences were found within the disturbed regime for either shoot or roots.

Eighty two OTUs were found considering all wheat samples (Supplementary Table S2). *Claroideoglomus* genus represented an average of 42% of the sequences, uncultured Glomeraceae around 41%, *Rhizophagus* 10%, *Acaulospora* 6%, Funneliformis around 2%, and *Scutellospora*, *Gigaspora*, *Paraglomus*, *Archaeospora*, *Entrophospora*, and *Dominikia* comprised less than 1% (Supplementary Table S2). AMF richness varied from 36 to 56 OTUs per sample. When comparing all OTUs from wheat, *O. compressus* and *L. rigidum*, regardless of the treatment, it was found that wheat had 24 OTUs that were not found in either of the previous hosts, but 40 OTUs were shared by the three species (Supplementary Figure S4).

**FIGURE 2:**
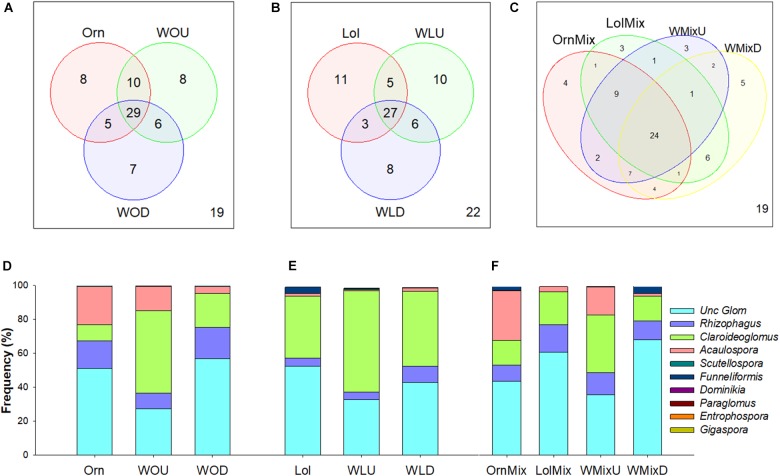
Shared and unique OTUs between *Ornithopus compressus*, *Lolium rigidum* and wheat (*Triticum aestivum*), and root AMF communities’ structure. **(A–C)** Venn diagrams from the three treatments, representing the number of unique and shared OTUs between the different hosts. **(D–F)** Frequencies of AMF taxa grouped by genera in the different hosts and treatments. OTUs with no match in the databases at genus level were considered as uncultured Glomeraceae. In all cases: Orn (*O. compressus*), Lol (*L. rigidum*), OrnMix (*O. compressus* from the mixture with *L. rigidum*), LolMix (*L. rigidum* from the mixture with *O. compressus*). Wheat: WOU (wheat after *O. compressus*, undisturbed), WOD (wheat after *O. compressus*, disturbed), WLU (wheat after *L. rigidum*, undisturbed), WLD (wheat after *L. rigidum*, disturbed), WMixU (wheat after *O. compressus* and *L. rigidum* grown in a mixture, undisturbed), WMixD (wheat after *O. compressus* and *L. rigidum* grown in a mixture, disturbed).

For each host combination separately, the number of OTUs shared between first hosts and wheat ranged between 24 and 29 (Figures 2A–C). For the system with *O. compressus* alone as first host, the number of unique OTUs (8) was the same between Orn and WOU. Ten OTUs were exclusive to these two groups, more than any of the other pairwise comparisons (Figures 2A–C). In the system with *L. rigidum* as first host (Figure 2B), Lol had more unique OTUs (11) than WLD (8) or WLU (10). Five OTUs were found only in Lol and WLU, and three OTUs were exclusive to Lol and WLD. In the system where both first hosts grew as a mixture (Figure 2C), nine OTUs were exclusive to OrnMix and WMixU. *L. rigidum* in the mixture (LolMix, Figure 2C) only had 1 OTU (*Claroideoglomus*) shared with WMixU, but shared six with WMixD (three *Claroideogmus* and three uncultured Glomeraceae).

Based on the frequencies of the AMF taxa on each system, WOU had a larger proportion of *Claroideoglomus* and *Acaulospora* than in the disturbed treatment (WOD) (Figure 2D). In the system with *L. rigidum* as first host, *Claroideoglomus* was more abundant in WLU than in Lol or WLD, whereas *Acaulospora* was almost absent in all (Figure 2E). For the mixture system (Figure 2F), the frequencies of AMF taxa showed a pattern more similar to OrnMix and WMixU (having, for example, more *Acaulospora*), and LolMix and WMixD; the first pattern was comparable to *O. compressus* grown on its own and also to WOU, and the latter to WOD (Figure 2D).

**FIGURE 3:**
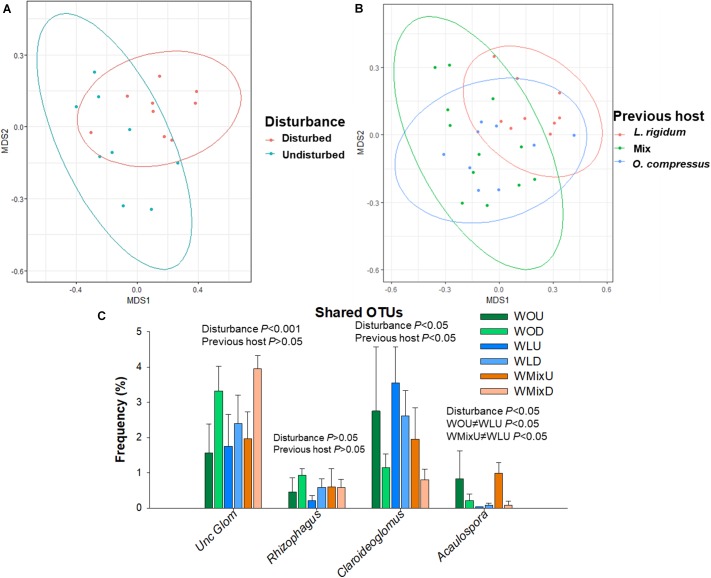
Effects of extraradical mycelium (ERM) disturbance and previous host on AMF communities’ composition in wheat (*Triticum aestivum*). **(A)** Effect of extra-radicular mycelia (ERM) network disruption on wheat AMF composition represented in multidimensional space (NMDS plot) considering the communities associated to wheat from the undisturbed and disturbed treatments. Ellipses represent ordination confidence intervals (95%), stress = 0.12. **(B)** NMDS plot of OTU composition in wheat + *O. compressus*+ *L. rigidum* on standardized data, showing that they form groups according to first host: *L. rigidum*, *O. compressus* or a mixture of both species. Ellipses represent ordination confidence intervals (95%), stress = 0.20. **(C)** Frequencies of shared AMF taxa grouped by genera in wheat. OTUs with no match in the databases at genus level were considered as uncultured Glomeraceae. Significant differences between treatments were assessed by Two-way ANOVA, with disturbance and first hosts as factors. WOU (wheat after *O. compressus*, undisturbed soil), WOD (wheat after *O. compressus*, disturbed soil), WLU (wheat after *L. rigidum*, undisturbed soil), WLD (wheat after *L. rigidum*, soil disturbed), WMixU (wheat after *O. compressus* + *L. rigidum*, undisturbed soil), WMixD (wheat after *O. compressus* + *L. rigidum*, disturbed soil).

PERMANOVA analysis revealed that, regardless of the previous host, wheat OTUs differed between intact and disturbed ERM (*F* = 4.2, *R^2^* = 0.21, *P* < 0.01) (Figure 3A). Furthermore, wheat, when analyzed together with *O. compressus* and *L. rigidum*, also formed clusters according to the first host groups (*O. compressus*, *L. ridigum* and the mixture of both) (*F* = 2.8, *R^2^* = 0.17, *P* < 0.01), which was corroborated by NMDS ordination (Figure 3B). This indicated that AMF communities of wheat, although highly influenced by ERM disturbance, were not independent of the AMF communities developed by the previous plants.

The analysis of the OTUs shared by the six wheat treatments revealed that OTUs belonging to uncultured Glomeraceae, *Claroideoglomus* and *Acaulospora* were significantly influenced by disturbance regime (Figure 3C). Previous host also had an effect on the relative abundance of *Claroideoglomus*, since wheat after *L. ridigum* had more *Claroideoglomus* than the other systems (*P* < 0.05) (Figure 3C). When analyzing only the undisturbed treatment, we found that wheat after *O. compressus* or after the mixture had a greater frequency of *Acaulospora* than wheat after *L. ridigum* (Figure 3C). These results are similar to those described above for *O. compressus* and *L. rigidum*.

The analysis of unique OTUs from the six wheat treatments showed that WOU had the greatest diversity of AMF genera (four OTUs, corresponding to four genera), while WLD had the smallest (1 OTU) (Supplementary Figure S3b).

### AMF β-Diversity in the Transition From First Host Species to Wheat

**FIGURE 4:**
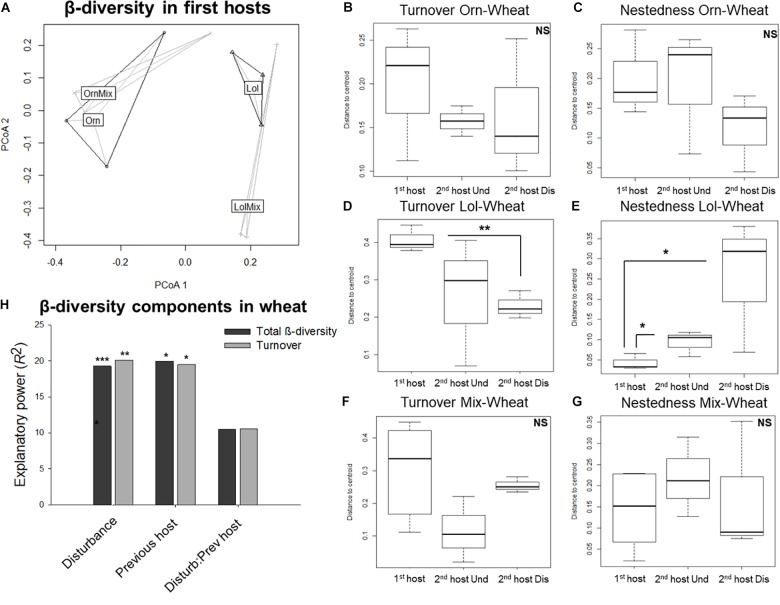
Arbuscular mycorrhizal fungi β-diversity in the transition from *Ornithopus compressus* and/or *Lolium rigidum* to wheat (*Triticum aestivum*). **(A)** AMF total β-diversity in the roots of first hosts in a principal coordinates space. **(B–G)** Boxplots showing the turnover and nestedness components of β-diversity between *O. compressus* and wheat **(B,C)**, *L. rigidum* and wheat **(D,E)**, and *O. compressus* + *L. rigidum* and wheat **(F,G)**, being wheat planted in either undisturbed (Und) or disturbed soil (Dis). Bold line represents median and the bottom and top of the box represent lower and upper quartiles. **(H)** Total amount of explanatory power (*R*^2^, scaled to 100) provided by disturbance, previous host species and their interaction on wheat global AMF β-diversity and components, as calculated based on PERMANOVA. Nestedness was null. ^∗∗∗^*P* < 0.001; ^∗∗^*P* < 0.01; ^∗^*P* < 0.05. NS, non-significant. In all cases: Orn, *O. compressus* alone; OrnMix, *O. compressus* neighbored by *L. rigidum*; Lol, *L. rigidum* alone; LolMix, *L. rigidum* neighbored by *O. compressus*.

Differences in AMF composition between first host species were clarified by considering AMF β-diversity in principal component analysis (Figure 4A). *L. rigidum* and *O. compressus* formed different clusters, whether they were planted singly or in a mixture.

We compared the multivariate homogeneity of dispersion (i.e., distance from objects to cluster centroid) in OTU turnover and nestedness between *O. compressus* and wheat (Figures 4B,C), *L. rigidum* and wheat (Figures 4D,E), and *O. compressus* + *L. rigidum* and wheat (Figures 4F,G). Only for *L. rigidum* as first host were there significant differences in the distances to the centroid (Figures 4D,E). Moreover, these differences were more obvious when the ERM was disturbed (Figures 4D,E). The analyses with Metastats indicated that the major distances obtained between the AMF community composition in *L. rigidum* and wheat in the disturbed treatment were mainly due to significant changes (*P* < 0.05, Supplementary Table S5) in the abundances of *Rhizophagus* OTUs (OTUs 18, 20, 31, 41), together with an *Acaulospora* OTU (OTU 49), and an uncultured Glomeraceae OTU (OTU 62). In the Undisturbed treatment, the differences were due to *Rhizophagus* OTUs (OTUs 26 and 47) and an uncultured Glomeraceae OTU (OTU 27).

The total amount of explanatory power (*R*^2^, scaled to 100) provided by disturbance, previous host species and their interaction on wheat global AMF β-diversity and components, was calculated based on PERMANOVA (Figure 4H). It showed that ERM disturbance and previous host had an effect on global β-diversity (*F* = 4.60, *R*^2^ = 0.19, *P* < 0.001 and *F* = 2.39, *R*^2^ = 0.20, *P* < 0.05, respectively) and on its turnover component (*F* = 4.85, *R*^2^ = 0.20, *P* < 0.01 and *F* = 2.36, *R*^2^ = 0.19, *P* < 0.01, respectively). Nestedness was found to have a null effect on variation between wheat treatments.

### Expression of Wheat Symbiosis-Related Genes

**FIGURE 5:**
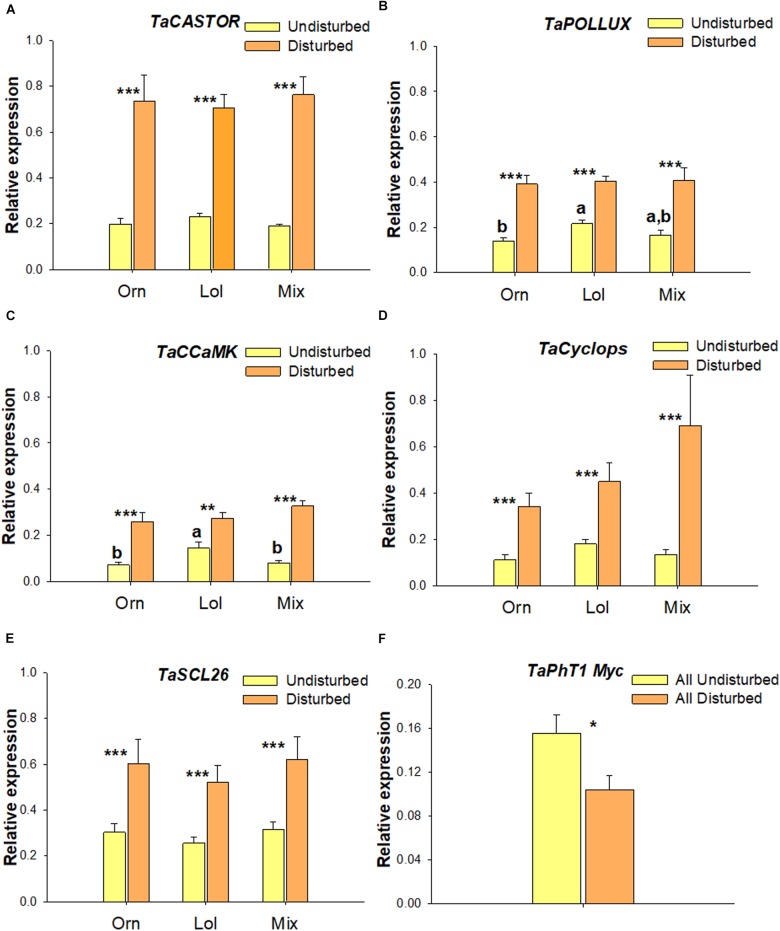
Expression of *CASTOR*, *POLLUX*, *CCaMK, Cyclops*, *SCL26* (*NSP2*) and *PhT1* Myc genes in wheat roots. Expression of wheat *TaCASTOR* **(A)**, *TaPOLLUX* **(B)** *CCaMK* **(C)**, *TaCyclops* **(D)**, *TaSCL26* (*NSP2*) **(E),** and *TaPhT1*Myc **(F)** as determined by RT-qPCR. ^∗^*P* < 0.05, ^∗∗∗^*P* < 0.001 indicate significant differences between undisturbed and disturbed treatments, and ^a,b^ between different previous AMF hosts (*P* < 0.05). Error bars indicate the standard error of the mean. Previous hosts: Orn, *O. compressus*; Lol, *L. rigidum*; Mix, *O. compressus*, *L. rigidum* grown in a mixture.

Five symbiosis-related *T. aestivum* genes – *TaCASTOR*, *TaPOLLUX*, *TaCCaMK*, *TaCyclops*, *TaSCL26* (*NSP2*) – were more strongly expressed in roots when the ERM network was disturbed, regardless of the previous host (*P* < 0.01) (Figures 5A–E). Moreover, within the undisturbed regime, *TaPOLLUX* and *TaCCaMK* showed significantly greater expression after *L. rigidum* than after *O. compressus* (*P* < 0.05) (Figures 5B,C). Expression of the specifically induced AMF phosphate transporter *TaPhT1* Myc was significantly greater in wheat roots from the undisturbed treatments than in the disturbed ones (*P* < 0.05) (Figure 5F).

## Discussion

### AMF Colonization Shows Host–Symbiont Preference

The strength of the relationship between symbiotic organisms, such as plants and their obligate biotrophic AMF colonizers, is expected to relate to the functional attributes of both partners and to favor relationships that better cope with environmental constraints. Defining the degree of preferential or non-random associations between AMF and plant host is crucial to understand the relative importance of both partners in driving this relationship.

In this study the AMF communities in soil and roots were dominated by members of the Glomerales, consistent with that of previous studies (e.g., Helgason et al., 2002; Öpik et al., 2006; Lumini et al., 2010). The AMF taxa found in the soil were similar following the growth of either of the first host species, and showed limited richness, though this might reflect the discrete sampling that was performed (prone to sampling only the most abundant species). Nevertheless, Bainard et al. (2014) reported previously that the plant host identity had minimal effect on the composition of the AMF community in the soil. Also a comparison of AMF taxa between soil mycelium, soil spores and colonized root propagules revealed a higher number of OTUs in roots than in soil, and that AMF taxa can be differentially allocated in these fractions (Varela-Cervero et al., 2015).

The community structure of AMF on yellow-serradella (*O. compressus*) differed greatly from that on ryegrass (*L. rigidum*), reflecting strong host–symbiont preferences. Yellow-serradella (Fabaceae) showed much greater overall OTU richness than did ryegrass (Poaceae). Differences in AMF type and richness between legumes and non-legumes have been described previously (Scheublin et al., 2004; Bainard et al., 2014; Brígido et al., 2017). This can partially be attributed to host taxonomic position, but factors such as ecosystem characteristics and functional specificities of the species cannot be disregarded.

Although our results were consistent with the hypothesis of host-specificity, the associated hypothesis on the existence of a neighbor effect was not supported. The simultaneous growth of the two species, yellow-serradella and ryegrass, had a minor effect on the network of symbionts established, only influencing the number of AMF genera among the unique OTUs present in the roots (just slightly greater when both species were grown together). Indeed, the reciprocal effect of neighboring hosts on root AMF communities seems to differ amongst species. For example, Hausmann and Hawkes (2009) reported that AMF communities on the grasses *Bromus hordeaceus* and *Nassella pulchra* were largely invariant, so that the presence of a neighbor was essentially neutral. In contrast, Yang et al. (2016) showed that mycorrhizal colonization of a legume tree was enhanced by legume neighbors but inhibited by grass neighbors. Our study showed almost no changes in the symbiotic community between yellow-serradella and ryegrass, possibly as a result of the different preferential host–symbiont associations. Thus, in AMF communities established on ryegrass there was a dominance of *Claroideoglomus*, whereas *Acaulospora* prevailed in those on yellow-serradella. A similar observation was reported by Brígido et al. (2017). This might relate to fungal colonization strategies and the development of ERM, which differ between AMF species (Hart and Reader, 2002; Schnepf et al., 2008; Powell et al., 2009) and could have implications for the delivery of functional benefits to the host plant.

### Shape of AMF Communities on Wheat Relates to Previous Host in an Undisturbed Environment

It is known that the order of plant establishment may differently filter AMF communities from the available species pool (Hausmann and Hawkes, 2010). The inheritance of a cohort of microorganisms gone through the plant filtering system can therefore provide a consortium that allows the plant to rapidly adjust to environmental conditions. The transition from yellow-serradella or ryegrass shaped wheat AMF consortia – when growing in the presence of an undisturbed mycelial – in a way that related to the identity of the previous host but did not relate to their taxonomic proximity (at least not at family level). Nevertheless, since wheat has evolved under cultivation, it is possible that this might modulate its mycorrhizal response in a different way from non-domesticated species (Martín-Robles et al., 2018). Domesticated plants have been bred to grow quickly and develop at a faster pace, and they probably exude different amounts and types of organic compounds in their roots, which can affect microbial communities (Pérez-Jaramillo et al., 2016).

While it was clear that wheat inherited AMF taxa from the previous host, the partitioning of root AMF β-diversity into its two components revealed a pattern that was not obvious from the analysis of genera frequency only: turnover and nestedness were significant in shaping AMF communities in the system with ryegrass as first host. However, differences between ryegrass and wheat, particularly wheat grown after soil disturbance, have been confined largely within the same taxa (*Rhizophagus*). It seems therefore that wheat and ryegrass specificities led to changes (AMF replacement or loss), but within closely related AMF species. Whether this is due to the taxonomical proximity of the two plant species (Poaceae) is currently unknown.

Soil disturbance has previously been related to changes in AMF composition in wheat roots (Brígido et al., 2017; Hartman et al., 2018). We also found great differences between OTU composition of wheat from disturbed and undisturbed treatments. This must relate to the main propagule source being spores and mycelia or colonized root fragments rather than a well-established ERM network. These sources are not equally effective at producing new infection units. Indeed, colonization initiated by an intact ERM starts earlier and develop faster (Goss and De Varennes, 2002; Brito et al., 2014; Alho et al., 2015) whereas the slower colonization from spores is linked to the need for germination to be initiated by signaling from the host plant (Garg and Chandel, 2010). Similarly to previous observations by Brito et al. (2014), we found significant differences in wheat growth between the undisturbed and disturbed regimes, but also between the yellow-serradella and ryegrass systems. Thus, it seems that AMF mediation of yellow-serradella-wheat facilitation (in the form of plant host benefit) occurred in an undisturbed environment. In fact, AMF have been shown to mediate plant interactions through a direct influence on plant biomass or by an indirect influence on plant photosynthesis and macronutrient acquisition (Yang et al., 2016).

### Co-planting of *O. compressus* and *L. rigidum* Reveals Wheat-AMF Non-random Associations

The mixture system (yellow-serradella and ryegrass planted as a mixture) generated in wheat an AMF frequency pattern, at the genera level, that more resembled that of yellow-serradella than of ryegrass. The difference specifically reflected the significantly greater frequency of *Acaulospora*. This suggests that, in an undisturbed environment, there was a non-random selection by wheat (and perhaps reciprocally by *Acaulospora*) from the available pool of AMF left after *L. rigidum* and *O. compressus*, and not simply passive root colonization by the most abundant taxa. Hence, AMF inheritance occurred (as observed by the single host species systems) but wheat-symbiont specificity emerges in the co-planting system. We see additional support for wheat-symbiont specificity as a large number of OTUs that did not occur in either *O. compressus* or *L. rigidum* were found in wheat. Among the six wheat treatments, overall β-diversity and the turnover component were influenced not only by the first host but also by soil disturbance. However, the contribution of nestedness or species loss to dissimilarities between wheat AMF communities was irrelevant, suggesting that AMF richness was mostly associated with plant species identity.

Complementary beneficial effects of different AMF can result in a more efficient exploitation of the soil nutrients. Facilitation was found in wheat from the yellow-serradella undisturbed treatment; by contrast, the effect declined when co-planting yellow-serradella and ryegrass, despite the AMF communities of wheat still resemble that of yellow-serradella. The reasons for this are unknown; however, functional consequences of the existence or abundance of specific OTUs (even within the same taxa) might be the explanation. In fact, genetic variability in different isolates of the same AMF species has been found to cause variations in plant growth (Koch et al., 2006). It has also been suggested that, as different plant species have distinct strategies for nutrient absorption, soil nutrients are used more effectively in the presence of a greater plant diversity, which may lead to a reduction in amount of nutrients available for uptake by the ERM for a specific plant (McKane et al., 2002).

### In Wheat, Symbiosis-Related Genes Respond to ERM Disturbance

Prior to and during colonization, molecular signals are exchanged between the two symbionts, leading to a stage-specific pattern of gene expression and hormonal signaling (Garg and Chandel, 2010). Our analysis of wheat root gene expression showed for the first time that ERM disturbance state had a considerable effect on five key symbiotic-related genes: *CASTOR, POLLUX*, *CCaMK*, *Cyclops*, and *SCL26* (*NSP2*) were expressed much more in roots from the disturbed than the undisturbed treatments. Soil disturbance implies the disruption of the mycelial network and thus the main propagule sources remaining were spores and colonized root fragments or mycelial fragments. These must re-enter the entire process of symbiosis establishment. On the other hand, in the undisturbed treatment a mycelial network was already established and apparently could easily colonize new roots; this easier colonization was further confirmed by the greater expression of the AMF-induced *PhT1* Myc gene in the systems with intact ERM. Also, the greater weight of wheat from the undisturbed treatments, regardless of the previous host indicates a more positive outcome of the cost-benefit balance for the symbiosis. The reduced activation of the genes in the undisturbed treatments certainly reflects the suitability of the existing ERM network. On the contrary, the greater expression of *CASTOR, POLLUX*, *CCaMK*, *Cyclops*, and *SCL26* in plants grown after soil disturbance might reflect an earlier stage in the symbiotic process. Consistent with our results, it has been observed that the requirements for CCaMK seem to be carefully regulated depending on the stage of root colonization (epidermal or cortical cell infection) (Liao et al., 2012).

We also found that the expression levels of *POLLUX* and *CCaMK* within roots from the undisturbed soil were significantly greater in wheat after *L. rigidum* than after *O. compressus*, which might relate to differential abundance of specific taxa. In fact, closely related AMF have been shown to alter transcriptional profiles of *Medicago trunculata* (Hohnjec et al., 2005). Moreover, Colard et al. (2011) showed that genetic exchange within the AMF *R. intraradices* species altered rice growth and the transcription of symbiotic genes. Kamel et al. (2017) found that the secretomes of *R. irregularis* and *G. rosea* when colonizing host plants differed according to host. Also genes related to stress responses have sequence polymorphisms between different *R. irregularis* isolates, which are likely to be related to its origin and natural environmental constraints and can have functional consequences for the host plant (Campos et al., 2015). Since disturbance and prior host changed the relative abundances of specific AMF taxa on wheat roots [for instance *Acaulospora* was highly sensitive to ERM disturbance, consistent with what has been described previously (Goss et al., 2017)], it remains to be seen if different AMF can induce changes in patterns of wheat gene expression and if or how this relates to the greater growth observed with *O. compressus* as previous host.

## Conclusion

Our results show that at least a part of wheat AMF consortia was shaped by the preceding plant host but without obliterating wheat-symbiont preferences; this was shown by wheat establishing preferential symbioses with specific AMF taxa on the undisturbed co-planting system. The ERM disturbance effect served therefore as proof-of-concept for wheat symbiotic preferences but also for wheat facilitation, as disrupted AMF communities were linked to a poorer growth. Root colonization starting from an intact indigenous and well-adapted AM fungal population therefore enhances the potential of AMF for promoting plant growth. Furthermore, we found that expression patterns of key symbiosis-related genes depended on ERM disturbance state and also partially on previous host, indicating that plant genetic program depends to some extent on the colonizing symbiont and on the type of propagule involved. Unraveling the molecular mechanisms beyond specific AMF-host interactions could provide a strategy to enhance performance of susceptible plants growing in adverse environments.

## Author Contributions

MC and TN conceived the study and experimental design. CC did the experiments and writing of the publication. CC and CB performed the bioinformatic data analyses. CC, TN, MC, CB, and MG did the interpretations. All authors read and approved the final manuscript.

## Conflict of Interest Statement

The authors declare that the research was conducted in the absence of any commercial or financial relationships that could be construed as a potential conflict of interest.

## Footnotes

1 http://genoscreen.fr/en/

2 http://www.premierbiosoft.com/
