# Supplementary material for: Symbiosis Specificity of the Preceding Host Plant Can Dominate but Not Obliterate the Association Between Wheat and Its Arbuscular Mycorrhizal Fungal Partners
Source: Front Microbiol. 2018 Nov 27;9:2920. doi: 10.3389/fmicb.2018.02920 (PMC6277769; doi:10.3389/fmicb.2018.02920)
Supplement: Supplementary file 1 [file Data_Sheet_1.PDF]

Frontiers in Microbiology **Supporting Information Fig. S1-S4 and Tables S1, S3, S4 and S5**

Article title: Symbiosis specificity of the preceding host plant can dominate but not obliterate the association between wheat and its arbuscular mycorrhizal fungal partners.

Authors: Catarina Campos, Mário Carvalho, Clarisse Brígido, Michael J. Goss and Tânia Nobre

The following Supporting Information is available for this article:

Fig. S1. Rarefaction analyses of arbuscular mycorrhizal fungi associated to different plant species (*Lolium rigidum*, *Ornithopus compressus*, and wheat (*Triticum aestivum*)).

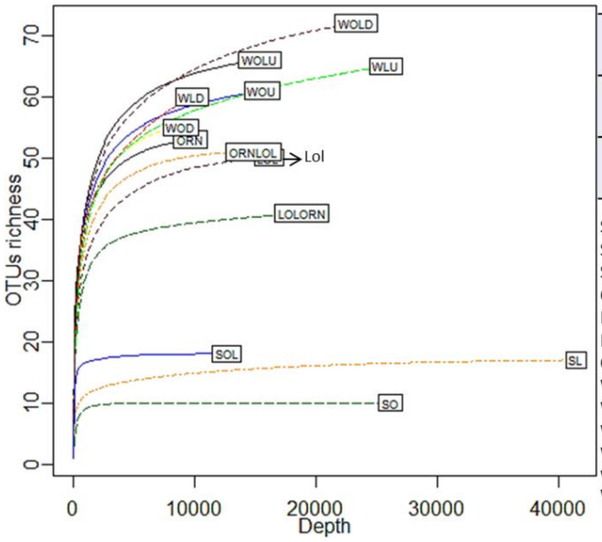

Slope values

SO	SL	SOL
2.51E-06	1.97E-04	2.19E-05

ORN	LOL	LOLORN	ORNLOL
3.44E-04	5.62E-04	2.69E-04	3.81E-04

WOU	WOD	WLU	WLD	WOLU	WOLD
6.28E-04	0.00E+00	8.33E-04	1.17E-03	7.18E-04	1.11E-03

SO – soil after *O. compressus*
SL – soil after *L. rigidum*
SOL – soil after *O. compressus* + *L. rigidum*
ORN - *O. compressus*
LOL - *L. rigidum*
LOLORN - *L. rigidum* (grown with *O. compressus*)
ORNLOL - *O. compressus* (grown with *L. rigidum*)
WOU – wheat grown after *O. compressus* undisturbed
WOD – wheat grown after *O. compressus* disturbed
WLU – wheat grown after *L. rigidum* undisturbed
WLD – wheat grown after *L. rigidum* disturbed
WOLU – wheat grown after *O. compressus* + *L. rigidum* undisturbed
WOLD – wheat grown after *O. compressus* + *L. rigidum* disturbed

Fig. S2. AMF taxa composition in soil and *Ornithopus compressus* and *Lolium rigidum* as first hosts. **a**) Pie chart for AMF taxa grouped by genera in soil. **b**) Non-metric multidimensional scaling (NMDS) plot of OTU composition in roots of first plant hosts (*O. compressus* + *L. rigidum*) and soil samples. Ellipses represent ordination confidence intervals (95 %), stress = 0.17.

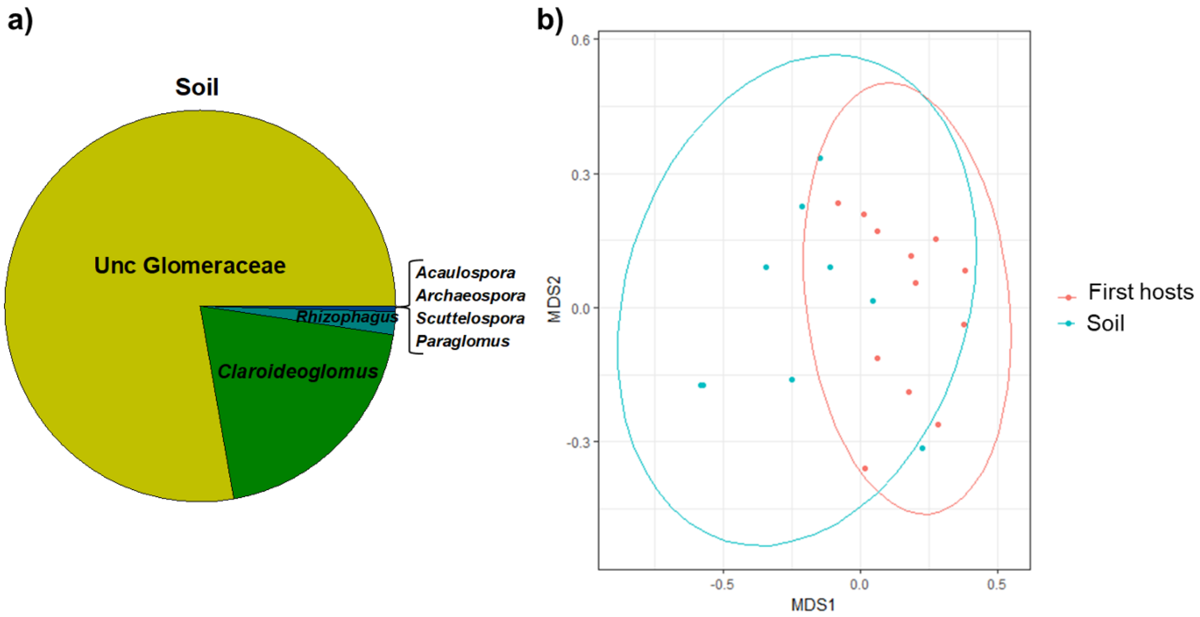

Fig. S3. a) Frequencies of unique OTUs in *Ornithopus compressus* (Orn) and *Lolium rigidum* (Lol) growing separately or in co-planting (Mix), and b) frequencies of unique OTUs amongst the six wheat treatments: WOU (wheat after *O. compressus*, undisturbed), WOD (wheat after *O. compressus*, disturbed), WLU (wheat after *L. rigidum*, undisturbed), WLD (wheat after *L. rigidum*, disturbed), WMixU (wheat after *O. compressus* and *L. rigidum* grown in a mixture, undisturbed), WMixD (wheat after *O. compressus* and *L. rigidum* grown in a mixture, disturbed).

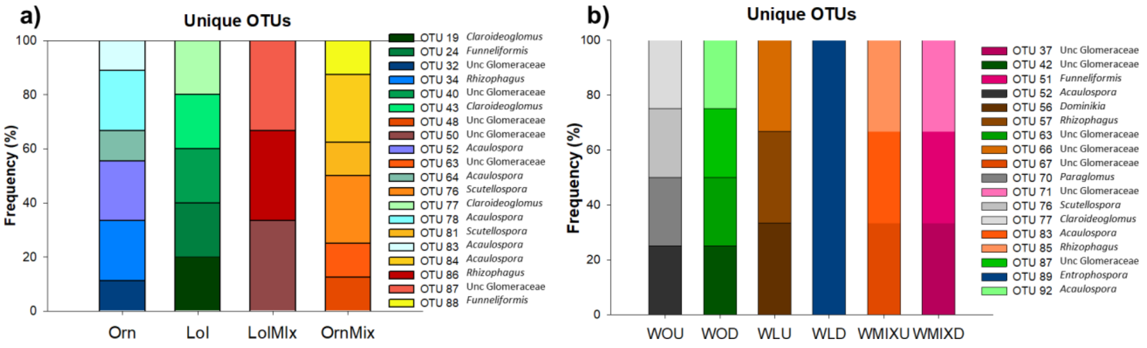

Fig. S4. Comparison of shared and unique OTUs between *Lolium rigidum*, *Ornithopus compressus*, and wheat (*Triticum aestivum*). A) Venn diagram from the three species representing the total number of unique and shared OTUs between the different hosts: Orn (*O. compressus*), Lol (*L. rigidum*) and wheat. **B**) Frequencies of unique OTUs grouped by genera in each host species. C) Comparison of frequencies of shared AMF grouped by genera between wheat, *O. compressus* (Orn) and *L. rigidum* (Lol). Significant differences between hosts for the same AMF genus were assessed by One-way ANOVA and are indicated by different letters ($P <$ 0.05).

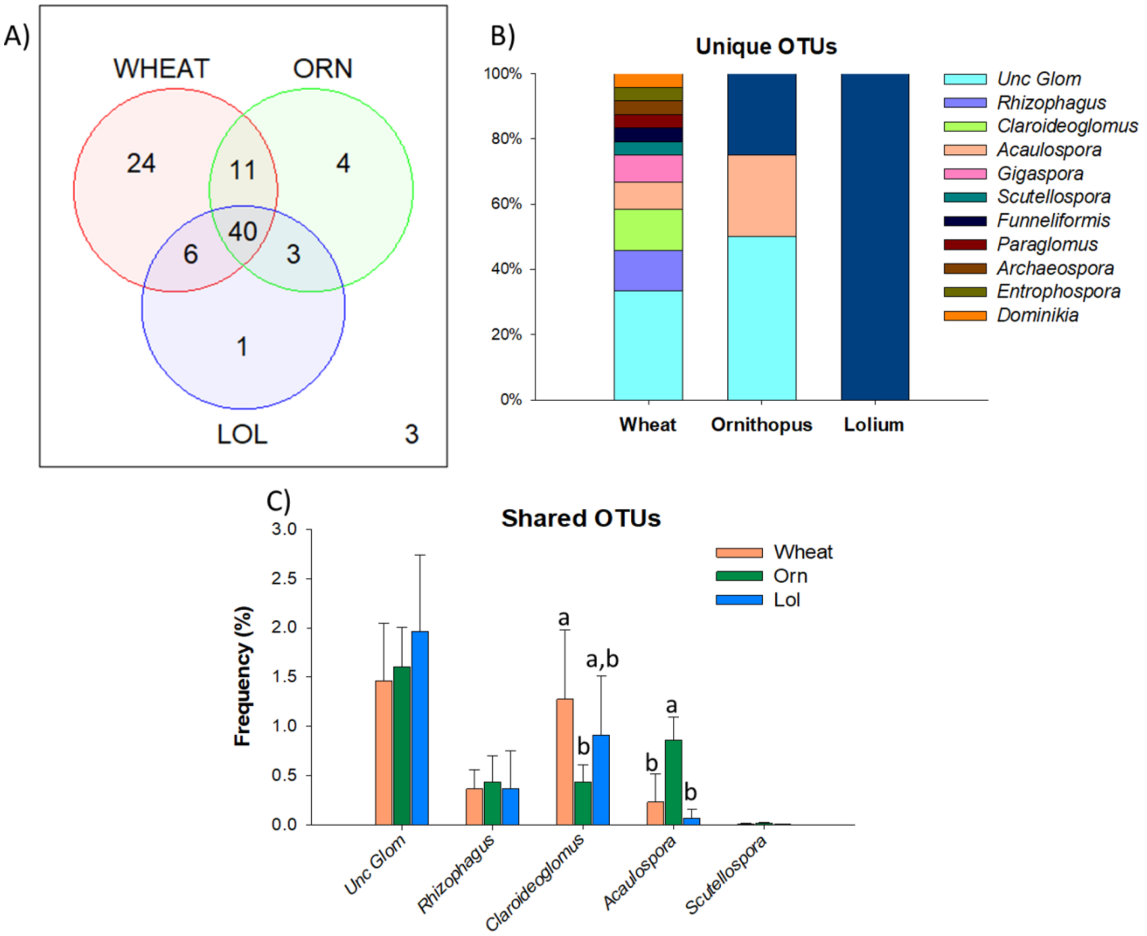

Table S1. Primers used in the analysis of wheat (Triticum aestivum) symbiosis-related genes by RT-qPCR.

**Gene**	**Forward sequence (5'→3')**	**Reverse sequence (5'→3')**	**Accession no Ensembl Plants/Ref**	**Size (bp)**	***E (%)***
GAPDH	ATTAAGGGTGGTGCCAAGAAG	CAGACTTGTATTCCTTCTCATTGAC	TRIAE_CS42_6DL_TGACv1_526670_AA1689480.1	94	95
Ta54227	CAAATACGCCATCAGGGAGAACATC	CGCTGCCGAAACCACGAGAC	Paolacci et al. (2009)	227	93
PhT1 Myc	AAGGTACTTGATCCAGCTTCTTG	CGAAGTTTGCGAAGAAGAATG	TRIAE_CS42_4DS_TGACv1_362305_AA1178710.1	143	93
CASTOR	CATGATGCTTGGTCTTGTGACTG	TTCAGTAAAGATCCCAACTTGTCAC	Traes_5BL_3A626410F	132	88
POLLUX	ACCTGGCTTGGCACAGATATG	GGCACAGCATCAGGGAATG	Traes_3AL_033375A71	129	102
CCaMK	GATTATGGATTTCGGTTTGAGTTC	ATGAAATGGTGGGCATCCG	Traes_1DL_C3C98B159.2	181	92
Cyclops	ATCTAGGTTGTCATCAGAGGCAG	GTTATTCGTGTATTCAGGAGGAAC	TRIAE_CS42_4AL_TGACv1_289135_AA0965550.1	124	101
SCL26 (NSP2)	AACTGCATACTCCACCAAGC	CCTCCTCCTCGTTCTTCTCC	TRIAE_CS42_4DL_TGACv1_343109_AA1129920.1	151	90

For each gene, accession number, amplicon size (bp) and amplification efficiency are indicated. The annealing temperature of all primer pairs is 60 °C.

Table S3. Indicator OTUs for *Ornithopus compressus* and *Lolium rigidum* obtained by IndVal analysis

**Host**	**IndVal**	***P*-value**	**OTU**	**Taxonomy**
O. compressus	0.89	**0.0070**	13	*Acaulospora* sp.
O. compressus	0.89	**0.0018**	49	*Acaulospora baetica*
O. compressus	0.88	**0.0020**	28	*Acaulospora ignota*
O. compressus	0.85	**0.0130**	9	*Acaulospora* sp.
O. compressus	0.69	**0.0450**	25	*Claroideoglomus etunicatum*
L. rigidum	0.84	**0.0030**	3	*Claroideoglomus* sp.

Table S4. Shoot and root fresh weight (FW) (g) of wheat (*Triticum aestivum*) in the different systems and disturbance regimes.

	Shoot FW (g) (mean ± SD)	Root FW (g) (mean ± SD)
**WOU**	**1.31 ^{a} ± 0.33**	**0.45 ^{a} ± 0.20**
**WOD**	**0.40 ^{c} ± 0.10**	**0.16 ^{c} ± 0.09**
**WLU**	**0.96 ^{b} ± 0.19**	**0.33 ^{ab} ± 0.16**
**WLD**	**0.41 ^{c} ± 0.08**	**0.09 ^{c} ± 0.05**
**WOLU**	**0.97 ^{b} ± 0.20**	**0.25 ^{b} ± 0.11**
**WOLD**	**0.42 ^{c} ± 0.05**	**0.18 ^{c} ± 0.06**

WOU - wheat grown after *O. compressus,* undisturbed;
WOD - wheat grown after *O. compressus,* disturbed;
WLU - wheat grown after *L. rigidum*, undisturbed;
WLD - wheat grown after *L. rigidum*, disturbed;
WOLU - wheat grown after *O. compressu*s + *L. rigidum,* undisturbed;
WOLD - wheat grown after *O. compressus* + *L. rigidum,* disturbed.

Table S5. Differentially abundant OTUs between the AMF communities of *Lolium rigidum* and wheat (*Triticum aestivum*), obtained by Metastats analysis.

	***P*-value**	**OTU# (see Table S1)**	**Taxonomy**
**Lol-WLD**	**0.0046**	OTU 18	*Rhizophagus intraradices*
**Lol-WLD**	**0.0170**	OTU 20	*Rhizophagus invermaium*
**Lol-WLD**	**0.0233**	OTU 31	*Rhizophagus* sp.
**Lol-WLD**	**0.0348**	OTU 41	*Rhizophagus irregularis*
**Lol-WLD**	**0.0401**	OTU 49	*Acaulospora baetica*
**Lol-WLD**	**0.0117**	OTU 62	Uncultured glomeraceae
**Lol-WLU**	**0.0050**	OTU 26	*Rhizophagus intraradices*
**Lol-WLU**	**0.0025**	OTU 27	Uncultured glomeraceae
**Lol-WLU**	**0.0000**	OTU 47	*Rhizophagus* sp.

Lol - *L. rigidum*; WLD - wheat grown after *L. rigidum*, disturbed treatment; WLU - wheat grown after *L. rigidum*, undisturbed treatment.
